# Simple Ways to Estimate Meningioma Volume: Can ABC- and SH-Derived Methods Be Used in Clinical Practice Reliably?

**DOI:** 10.1155/2021/9712287

**Published:** 2021-08-23

**Authors:** Dongdong Xiao, Jun Liu, Tingting Hu, Burkutally Mohammad Shah Nayaz, Xiaobing Jiang, Fangcheng Zhang, Pengfei Yan

**Affiliations:** ^1^Department of Neurosurgery, Union Hospital, Tongji Medical College, Huazhong University of Science and Technology, Wuhan 430022, China; ^2^Department of Neurosurgery, Taihe Hospital, Hubei University of Medicine, Shiyan 442000, China

## Abstract

**Background:**

There is a clinical demand for rapid estimation of meningioma volumes. Our objective was to assess the accuracy of three ABC-derived and three SH-derived formula methods on volume estimation of meningiomas.

**Methods:**

The study group comprised 678 patients treated at our department for histopathologically proven intracranial meningiomas. For each patient, tumor volumes were independently measured using six formula methods as well as planimetry. Maximum tumor diameter and ellipsoidity were also recorded. Volumes were compared using descriptive statistics, correlation analysis, and consistency analysis.

**Results:**

Among all methods assessed, 2/3SH and 1/2ABC outperformed the others. No significant differences were found between volumes obtained by the two methods and those of planimetry (*p* > 0.05). Spearman rank-correlation coefficients (*r*_*s*_) were 0.99 for both methods (*p* < 0.01), and ICC were 0.99 and 0.98, respectively. In Bland-Altman plot, most data points lay inside the limit of agreement. Overall, 2/3SH overestimated tumor volumes by 1.29%, and estimation errors in 93.66% cases were within 20%; 1/2ABC overestimated tumor volumes by 5.36%, and estimation errors in 93.51% cases were within 30%. The performance of 2/3SH and 1/2ABC in small-volume meningiomas was slightly worse, especially for 1/2ABC. Correlations between ellipsoidity and percentage errors of 2/3SH and 1/2ABC were weak (*r*_*s*_ = −0.06 and −0.24, respectively). Despite a significant correlation between maximum tumor diameter and planimetric volume (*r*_*s*_ = −0.96), volumes could vary significantly for a given diameter.

**Conclusions:**

Formula methods 2/3SH and 1/2ABC can estimate meningioma volumes with decent accuracy. Compared with the 1/2ABC method, the 2/3SH method showed slightly better performance, especially in small-volume meningiomas. Ellipsoidity is not a suitable parameter to predict estimation error, and maximum tumor diameter is not a reliable surrogate for actual meningioma volume.

## 1. Introduction

With an incidence of 8.58/100000, meningioma is the most frequent primary tumor of the central nervous system, accounting for 37.6% of tumors overall [1]. A considerable percentage of meningiomas are actually diagnosed incidentally during routine radiographic examinations [2]. Given the slow-growing and indolent nature of most meningiomas, physicians usually face the dilemma of whether to operate or just follow the wait-and-see policy, especially for those who are asymptomatic or elderly [3, 4]. Tumor volume plays a pivotal role in this decision-making process. For instance, large tumors can increase the intracranial pressure substantially, making surgery an optimal treatment choice. Besides, tumor volume is reported to correlate with histological aggressiveness; meningiomas with larger volumes are more often diagnosed as high-grade [5, 6]. Considering the fact that more aggressive treatment should be considered for patients with high-grade meningiomas, tumor volume might serve as a good indicator in this respect. In addition, tumor volume is closely related to stereotactic radiosurgery (SRS) [7–9]. Bloch et al. found that the tumor control rates of SRS may decrease with increased volume of meningiomas and that radiation toxicity increased with increasing tumor volume [7]. Mansouri et al. indicated that tumor volume impacted such factors as the maximal safe deliverable SRS dose and the dose reduction necessary to avoid radiation toxicity, thus affecting the overall treatment outcome [8]. Therefore, it can be used as an effective parameter to select patients suitable for SRS.

There are different methods available to estimate tumor volume. So far, planimetry is the most accurate one. To perform planimetric measurement, traditionally, physicians need to segment the tumor on each image slice and then calculate the cumulative volume enclosed by the segmentation. Although accurate, it is usually time- and labor-intensive. With recent advances in imaging algorithms, some software can now segment tumors semiautomatically: physicians need to only manually outline the tumor on a small number of image slices, and the software could automatically identify the tumor on the slices in between (interpolated segmentation). This function has greatly benefitted clinical work, but it is not without limitations. The major one is that electronic DICOM data is necessary for such measurement; in certain work settings, however, such DICOM data may not be accessible. In such cases, simple measurement methods that can be performed directly with images physically stored in printed form are desired. Among all methods proposed so far, an ellipsoid-based method named 1/2ABC is mostly studied/used. This method was initially proposed for intracerebral hemorrhage; with time, it was also used to calculate volumes of other intracranial lesions, including epidural hematoma, subdural hematoma, vestibular schwannoma, glioma, infarction, and arteriovenous malformation [10–16]. Some similar methods have also been proposed, such as 2/3ABC, 1/2SH, and 2/3SH [17–20]. These formula methods share the same advantages—they are easy to understand, quick to perform, and have low interrater and intrarater variations. However, the performance of these methods on the volume estimation of meningiomas has not yet been systematically investigated. If evidence was found that any of them does provide acceptable volume estimations, clinical work would be much facilitated.

In this study, we evaluated the performance of six different formula methods on volume estimation of meningiomas. Besides, we also validated their accuracies in the subset of small-volume tumors. Moreover, as maximum tumor diameter was occasionally used as a surrogate for actual meningioma volume in previous reports, we assessed the correlation between the two measurements.

## 2. Patients and Methods

### 2.1. Patient Population

This study was approved by our institutional review board, and written informed consent was waived due to the retrospective nature of the study and the anonymity of the data. We identified surgical patients treated at our department for histopathologically proven intracranial meningiomas between March 2013 and January 2020. Patients were included if they met the following criteria: (1) underwent MR imaging within two weeks before surgery; (2) no more than one tumor present on MR images; (3) number of tumor-bearing MRI slices > 4; (4) no noticeable motion artifacts found; (5) relevant clinical data available in the electronic medical records. For each patient, demographic variables, including age, sex, tumor location, and WHO grade, were collected. Scans were obtained with one of the two machines: Siemens Magnetom Verio 3-T and Siemens Magnetom Avanto 1.5-T, each with an 8-channel radiofrequency coil. The MRI protocol used the following parameters: field of view, 230 × 230 mm; matrix size, 512 × 512; slice thickness, from 4 to 5 mm; and flip angle, 90°. The repetition time (TR)/echo time (TE) for the T1-weighted sequence (T1WI), the T2-weighted sequence (T2WI), and the fluid-attenuated inversion recovery (FLAIR) sequence was 500/8.4 ms, 9000/89 ms, and 9000/105 ms, respectively. Contrast-enhanced T1-weighted images (CE-T1WI) were used for tumor segmentation; they were obtained in the sagittal and axial planes after intravenous administration of 0.2 mL/kg gadopentetate dimeglumine. All these images were stored in Picture Archiving and Communication Systems (PACS) and could be viewed by physicians.

### 2.2. Volume Calculation

**Figure 1:**
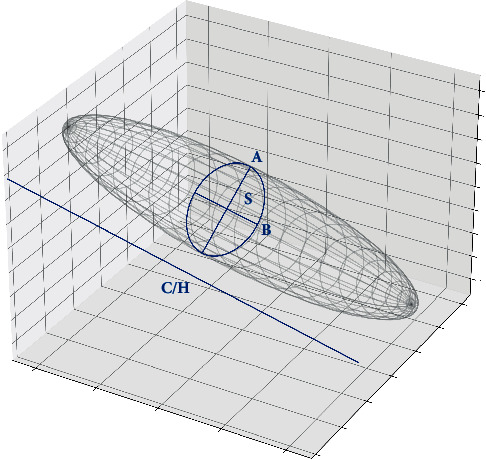
Illustration of the formula methods. For volume estimation, the axial MRI slice with the maximum tumor area is selected (the tumor area in this slice is denoted as S); the maximum tumor length in this slice is denoted as A, and the maximum tumor width (perpendicular to A) in this slice is denoted as B. Tumor height (slice thickness, the number of all tumor-bearing axial slices) is denoted as C or H. Then, tumor volume is estimated using one of the following six formula methods: 1/3 × ABC, 1/2 × ABC, 2/3 × ABC, 1/3 × SH, 1/2 × SH, and 2/3 × SH.

Firstly, manual segmentation of each meningioma was performed by one author (JL) with ITK-SNAP (version 3.8.0, University of Pennsylvania) and was later rechecked and modified by another author (PFY) to ensure accuracy [21]. The software could automatically give the volume of each tumor by calculating voxel volume inside the segmentation (planimetry). According to the rationale behind this method, it is supposed to be able to provide the most accurate volume estimation with MRI; therefore, it was used as the gold standard in this study. Next, we obtained tumor volume estimations using six different formula methods (including three ABC-derived methods and three SH-derived methods). For calculating the volumes, the axial MRI slice with the maximum tumor area was selected (the tumor area in this slice was denoted as S); the maximum tumor length in this slice was denoted as A, and the maximum tumor width (perpendicular to A) in this slice was denoted as B. Tumor height (slice thickness multiplied by the number of all tumor-bearing axial slices) was denoted as C or H (Figure 1). Then tumor volume was calculated using the following six formula methods: 1/3ABC, 1/2ABC, 2/3ABC, 1/3SH, 1/2SH, and 2/3SH. All these methods have either been used clinically or been studied in previous reports for their efficacy in estimating lesion volumes. To avoid possible errors introduced by human raters, an in-house script written in Python (version 3.8.3) was used to automatically detect and measure the parameters S, A, B, and C/H.

**Figure 2:**
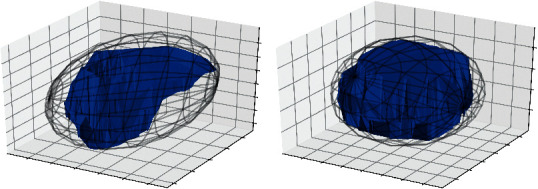
Illustration of the concept “ellipsoidity.” Ellipsoidity is defined as the volume ratio of the meningioma to its minimal bounding ellipsoid, which ranges between 0 and 1. A higher value indicates an ellipsoid-like/regular shape, and a lower value indicates an irregular shape. Here, we present two real cases in our dataset to illustrate this concept: tumor A has an ellipsoidity of 0.49, and tumor B has an ellipsoidity of 0.79.

Maximum tumor diameter was defined as the largest pairwise Euclidean distance between tumor surface in axial/coronal/sagittal plane. It was determined using Python's library PyRadiomics (version 3.0). In addition, we introduced the concept of “ellipsoidity,” which was defined as the volume ratio of the tumor to its minimal bounding ellipsoid. It ranged between 0 and 1; a higher value indicated an ellipsoid-like/regular shape, and a lower value indicated an irregular shape (Figures 2(a) and 2(b)).

### 2.3. Statistical Analysis

Statistical analysis was carried out using the MedCalc package (version 19.3), and *p* values of less than 0.05 were regarded as statistically significant [22]. Statistical figures were created using the plotting library Matplotlib (version 3.2.2) and Seaborn (version 0.10.1). Continuous variables were expressed as medians with interquartile ranges (IQR), and categorical variables were expressed as numbers with percentages. The data normality was checked by Shapiro-Wilk or Kolmogorov-Smirnov tests. Student's *t*-test or Mann-Whitney *U* test was used to assess volume differences between methods, based on data distributions. The correlations between the formula methods and the planimetry were examined using scatter plot and Spearman rank-correlation coefficient (*r*_*s*_). Subsequently, intraclass correlation coefficient (ICC) and Bland-Altman plot were used to evaluate the consistency between them. The volume estimation errors were evaluated using four indexes: volume difference (VD), percentage volume difference (PVD), absolute volume difference (AVD), and absolute percentage volume difference (APVD). VD was defined as [volume of formula method-planimetric volume], PVD was defined as [(volume of formula method−planimetric volume)/planimetric volume × 100%], and AVD and APVD were defined as the absolute values of the above two indexes.

## 3. Results

### 3.1. Study Sample

A total of 678 patients were included in the study, out of which 589 patients (86.87%) suffered from low-grade meningiomas, and 89 patients (13.13%) suffered from high-grade meningiomas. 173 patients (25.52%) were male, and 505 patients (74.48%) were female, giving a male to female ratio of 1 : 2.92. The median patient age at the time of surgery was 53 years (IQR 47 to 60 years). Tumor locations were as follows: 198 patients (29.20%) in convexity, 182 patients (26.84%) in falx or parasagittal, 51 patients (7.52%) in anterior cranial fossa, 103 patients (15.19%) in middle cranial fossa, 110 patients (16.22%) in posterior cranial fossa, and 34 patients (5.01%) in other sites.

### 3.2. Accuracy of Different Formula Methods

**Figure 3:**
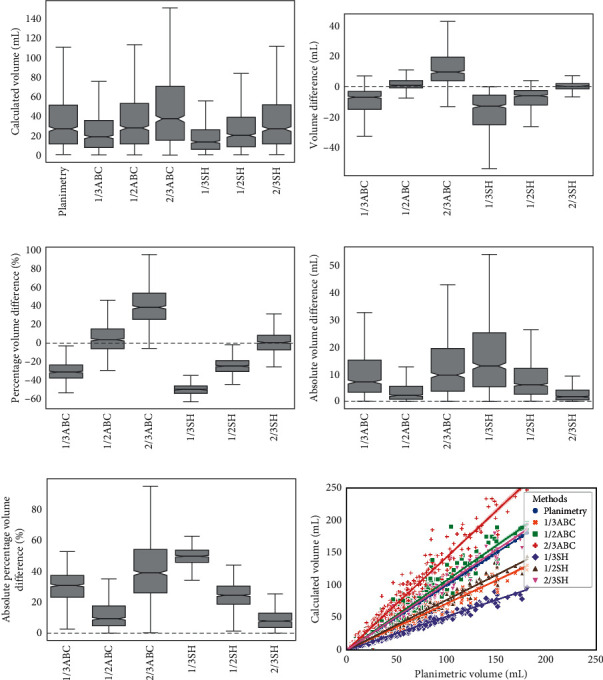
Comparisons of different methods. (a) Volumes calculated by planimetry and the formula methods. (b) Volume differences (VD) between planimetry and the formula methods. (c) Percentage volume differences (PVD) between planimetry and the formula methods. (d) Absolute volume differences (AVD) between planimetry and the formula methods. (e) Absolute percentage volume differences (APVD) between planimetry and the formula methods. (f) Scatter plot of planimetry and the formula methods.

The volume medians of planimetry, 1/3ABC, 1/2ABC, 2/3ABC, 1/3SH, 1/2SH, and 2/3SH were 26.65 mL (IQR 11.38 to 51.21 mL), 18.48 mL (IQR 7.57 to 35.29 mL), 27.72 mL (IQR 11.35 to 52.94 mL), 36.95 mL (IQR 15.13 to 70.58 mL), 13.35 mL (IQR 5.71 to 25.75 mL), 20.02 mL (IQR 8.57 to 38.63 mL), and 26.70 mL (IQR 11.43 to 51.50 mL), respectively (Table 1; Figure 3(a)). Using the Mann-Whitney *U* test, statistically significant differences were found between the planimetric volume and the volumes obtained by formulas 1/3ABC, 2/3ABC, 1/3SH, and 1/2SH (*p* < 0.01). No significant differences were found between volumes obtained by formula 1/2ABC or 2/3SH and those of planimetry (*p*=0.52 and 0.97, respectively).

The VD, PVD, AVD, and APVD between any random pairs of the six formula methods were statistically significant (*p* < 0.01). The VD medians of 1/2ABC and 2/3SH were 0.64 mL (IQR −0.94 to 3.82 mL) and 0.10 mL (IQR −1.61 to 1.88 mL), respectively. The PVD medians of 1/2ABC and 2/3SH were 3.87% (IQR −5.80 to 15.38%) and 0.82% (IQR −7.08 to 8.45%), respectively. The AVD medians of 1/2ABC and 2/3SH were 2.17 mL (IQR 0.79 to 5.61 mL) and 1.73 mL (IQR 0.60 to 4.14 mL), respectively. The APVD medians of 1/2ABC and 2/3SH were 9.28% (IQR 4.75 to 17.49%) and 7.79% (IQR 3.62 to 12.83%), respectively. Figures 3(b)–3(e) demonstrate that VD, PVD, AVD, and APVD of 1/2ABC and 2/3SH were closer to 0 than the other groups, indicating their better ability for volume estimation.

As the scatter plot in Figure 3(f) shows, all the formulas roughly followed linear relationships. Compared with the other four lines, the regression lines of 2/3SH and 1/2ABC lay closer to that of planimetry. Spearman rank-correlation coefficients of the three ABC-derived formulas and the three SH-derived formulas were both 0.99 (*p* < 0.01), suggesting strong correlations between the formula methods and planimetry.

**Figure 4:**
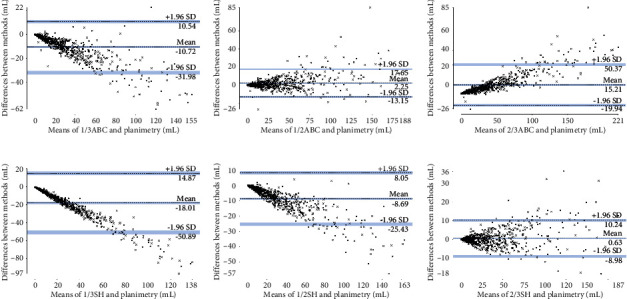
Bland-Altman plots of each formula method. In each plot, most data points are found to lie inside the limit of agreement (LoA). When considering the percentage of data points outside the LoA, the order from smallest to largest is 1/2ABC (4.87%), 2/3SH (5.01%), 1/3ABC (5.02%), 1/2SH (5.46%), 1/3SH (6.05%), and 2/3ABC (6.20%).

The order of ICCs from largest to smallest was 2/3SH (0.99), 1/2ABC (0.98), 1/2SH (0.96), 1/3ABC (0.94), 2/3ABC (0.91), and 1/3SH (0.81).  Figure 4 is the Bland-Altman plot of each formula method. Most data points lay inside the limit of agreement (LoA), indicating the general agreement between the formula methods and planimetry. When considering the percentage of data points outside the LoA, the order from smallest to largest was 1/2ABC (4.87%), 2/3SH (5.01%), 1/3ABC (5.02%), 1/2SH (5.46%), 1/3SH (6.05%), and 2/3ABC (6.20%). Taking all the above analysis into consideration, we can reasonably conclude that, among the six formula methods, 1/2ABC and 2/3SH outperformed the others and could be used for better volume estimation of meningiomas. Furthermore, if we define APVD <10% as clinically acceptable, then 1/2ABC and 2/3SH could provide reliable estimations in 52.80% and 63.42% of cases, respectively; if we define the criterion as 20%, the corresponding diagnostic accuracies were 79.65% and 93.66%, respectively; and if we raise the criterion to 30%, the corresponding diagnostic accuracies increased to 93.51% and 97.79%, respectively (Table 2).

### 3.3. Accuracy Validation of 1/2ABC and 2/3SH in Small-Volume Tumors

**Figure 5:**
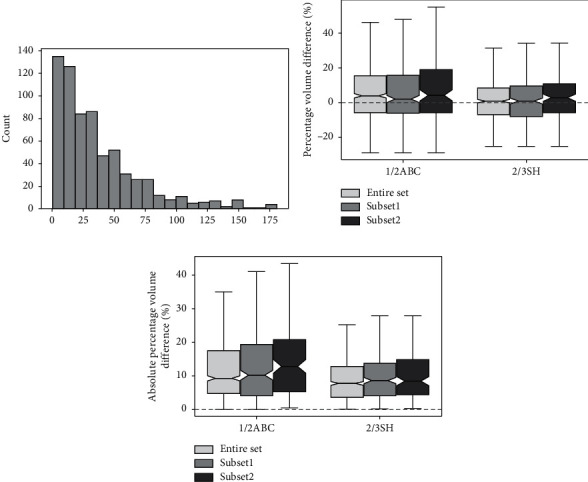
Performance comparison of 1/2ABC and 2/3SH in the three datasets. (a) Histogram showing the distribution of values of tumor volumes. (b) Comparison of percentage volume differences (PVD) in the three datasets. (c) Comparison of absolute percentage volume differences (APVD) in the three datasets.

We further validated the performance of 1/2ABC and 2/3SH in estimating volumes of small meningiomas. All cases were first graphed with a histogram to evaluate the distribution of the data. As Figure 5(a) shows, tumor volumes of 0–10 mL and 10–20 mL were in the two groups with the largest number of patients, accounting for 21.39% and 19.91% of all cases, respectively. Based on this information and real clinical practice, we defined small-volume tumors as either <10 mL (subset 1) or < 20 mL (subset 2).

The total number of patients in subset 1 was 145 (21.39%). In this subset, the VD medians of 1/2ABC and 2/3SH were 0.16 mL (IQR −0.19 to 0.89 mL) and 0.08 mL (IQR −0.23 to 0.57 mL), respectively. The PVD medians of 1/2ABC and 2/3SH were 4.15% (IQR −5.91 to 19.03%) and 2.81% (IQR −5.85 to 10.89%), respectively. The AVD medians of 1/2ABC and 2/3SH were 0.59 mL (IQR 0.18 to 1.16 mL) and 0.40 mL (IQR 0.16 to 0.84 mL), respectively. The APVD medians of 1/2ABC and 2/3SH were 12.86% (IQR 5.15 to 20.82%) and 8.48% (IQR 4.40 to 14.88%), respectively. If we define APVD <10% as clinically acceptable, then 1/2ABC and 2/3SH could provide reliable estimations in 43.45% and 58.62% of cases, respectively; if we define the criterion as 20%, the corresponding diagnostic accuracies were 72.41% and 88.97%, respectively; and if we define the criterion as 30%, the corresponding diagnostic accuracies increased to 88.28% and 94.48%, respectively. Spearman rank-correlation coefficients of the two methods were 0.93 and 0.97, respectively; ICCs of the two methods were 0.89 and 0.95, respectively.

The total number of patients in subset 2 was 280 (41.30%). In this subset, The VD medians of 1/2ABC and 2/3SH were 0.14 mL (IQR −0.49 to 1.16 mL) and 0.05 mL (IQR −0.68 to 0.66 mL), respectively. The PVD medians of 1/2ABC and 2/3SH were 1.99% (IQR −6.24 to 15.73%) and 0.86% (IQR −8.05 to 9.63%), respectively. The AVD medians of 1/2ABC and 2/3SH were 0.81 mL (IQR 0.30 to 1.80 mL) and 0.67 mL (IQR 0.22 to 1.44 mL), respectively. The APVD medians of 1/2ABC and 2/3SH were 10.22% (IQR 4.13 to 19.38%) and 8.63% (IQR 4.12 to 13.78%), respectively. If we define APVD <10% as clinically acceptable, then 1/2ABC and 2/3SH could provide reliable estimations in 49.64% and 58.93% of cases, respectively; if we define the criterion as 20%, the corresponding diagnostic accuracies were 76.43% and 90.36%, respectively; and if we raise the criterion to 30%, the corresponding diagnostic accuracies increased to 91.43% and 96.43%, respectively. Spearman rank-correlation coefficients of the two methods were 0.95 and 0.97, respectively; ICCs of the two methods were 0.93 and 0.97, respectively.

**Figure 6:**
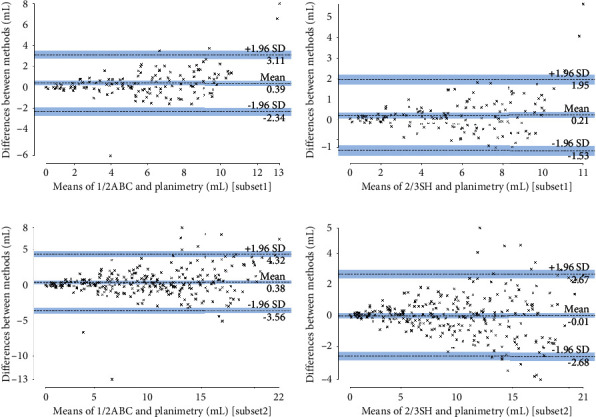
Bland-Altman plots of 1/2ABC and 2/3SH in subset 1 and subset 2. Similar as in the entire dataset, most data points also lie inside the limit of agreement (LoA). The percentages of data points outside the LoA range from 2.07 to 6.07%.

Figures 5(b) and 5(c) are boxplots demonstrating the estimation accuracy of 1/2ABC and 2/3SH in the three datasets. As Figure 5(c) shows, the general performance in subset 1 and subset 2 was slightly worse, especially for 1/2ABC. For 2/3SH, all relevant parameters, including the minimum, first quartile, median, third quartile, and maximum, did not differ much among the three datasets. In Bland-Altman plot, most data points also lay inside the LoA (Figure 6) For 1/2ABC and 2/3SH in subset 1, the percentage of data points outside the LoA was 3.45% and 2.07%, respectively. In subset 2, the corresponding values were 3.93% and 6.07%, respectively. Based on the above analysis, we can see that 2/3SH was more reliable in measuring small-volume meningiomas than 1/2ABC.

### 3.4. Correlation between Tumor's Ellipsoidity and Measurement Error

**Figure 7:**
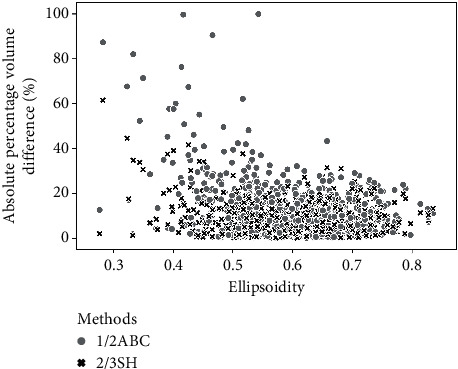
Correlation between tumor's ellipsoidity and estimation error. Scatter plot of ellipsoidity and absolute percentage volume difference (APVD), demonstrating a weak negative correlation; the corresponding Spearman rank-correlation coefficients for 1/2ABC and 2/3SH are −0.24 and −0.06, respectively.

We analyzed the correlations between ellipsoidity and APVD of 1/2ABC and 2/3SH. Although a negative trend could be seen in the scatter plot, the corresponding *r*_*s*_ were low, being −0.24 and −0.06, respectively (Figure 7). Thus, “ellipsoidity” defined in this study is not a good index to predict the potential estimation accuracies of these methods.

### 3.5. Correlation between Maximum Tumor Diameter and Tumor Volume

**Figure 8:**
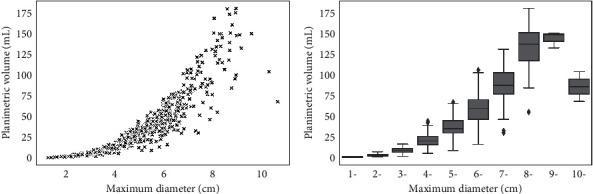
Correlation between maximum tumor diameter and planimetric volume. (a) Scatter plot of maximum tumor diameter and planimetric volume, a clear positive correlation can be noted. (b) Meningiomas grouped based on maximum tumor diameter (with one-centimeter intervals). With a given maximum diameter, the corresponding tumor volumes could vary much and vice versa.

The correlation between the maximum diameter and tumor volume was significant (*r*_*s*_ = 0.96, Figure 8(a)). We further divided the maximum diameters into ten groups with one-centimeter intervals; the corresponding volume distribution of each group can be seen in Figure 8(b). With a given maximum diameter, the corresponding tumor volumes could vary much. This discrepancy increased from group one to group eight and decreased in groups nine and ten. Taking group eight for example, the minimum volume was 55.51 mL, whereas the maximum volume was 180.80 mL; the range reached 125.29 mL. With a given tumor volume, the corresponding diameters could also vary. Therefore, although the two measurements correlated closely, maximum diameter is not a reliable surrogate for actual meningioma volume in clinical and research settings.

## 4. Discussion

As we discussed earlier, volume measurement plays a pivotal role in the management of patients with meningiomas. Many medical image software types now provide the function of semiautomatic segmentation, with which segmentation could be obtained in a few steps. However, this method might not be practical when digital copies of the DICOM images are not accessible. This is often encountered in outpatient settings, where hard-copy images in printed form are usually the only material available for diagnosis. In these situations, a simple measurement method that can be performed directly on the printed images would facilitate clinical decision-making. This demand could possibly be met with formula methods analyzed here. Among various options available, the one most often used is 1/2ABC, whose volume is obtained from ellipsoid approximation. This method has been assessed and validated in several intracranial lesions. However, till now, only a few reports have described its actual performance on meningiomas. Ishi et al. analyzed 83 meningiomas and divided them into skull base and nonskull base [23]. They acknowledged a significant correlation between the two methods, but the 1/2ABC tended to overestimate tumor volume, particularly for those located in the middle skull base. In a recent publication by Opalak et al., 146 follow-up images of 29 meningiomas were reviewed, and a good correlation between 1/2ABC and planimetry was found; the mean volume difference between methods was 0.2 mL, and the corresponding ICC was 0.95 [24]. Similarly, in the present study, results showed that 1/2ABC generally correlated well with planimetry. 1/2ABC overestimated tumor volume by 2.25 mL (or 5.36%) on average; if only absolute values were considered, the mean volume difference was 4.49 mL (or 13.10%). Estimation errors in 79.65% of cases were within 20% and in 93.51% of cases were within 30%. This degree of accuracy in general is roughly acceptable. This may be attributed to the relatively regular shape in most cases. In other tumor types whose shapes vary even more markedly, the estimation errors should be more prominent.

Another class of formula methods we evaluated was SH. As the 1/2ABC was likely to overestimate lesion volume in some cases, attempts were made to improve the accuracy by previous investigators. Some proposed using SH-derived formula methods, such as the 2/3SH and the 1/2SH [17–20]. In the publications by Shen et al., the 2/3SH was shown to be able to estimate volumes of epidural, subdural, and intracerebral hematomas with good accuracy (with PVD being nearly 1%, <1%, and 2%, respectively) [17, 18]. Zhao et al., in a recent paper, evaluated the performance of seven formula methods on volume calculation of spontaneous intracerebral hemorrhage. Through detailed analysis, they concluded that the accuracy of the 1/2SH and the *π*/6SH was satisfactory [19]. Inspired by these works, we included three SH-derived methods in our analysis. Results showed that, among the three methods, the 2/3SH performed better. It overestimated tumor volume by 0.63 mL (or 1.29%) on average; if only absolute values were considered, the mean volume difference was 3.02 mL (or 8.98%). Estimation errors in 63.42% cases were within 10% and in 93.66% cases were within 20%. The overall estimation accuracy of the 2/3SH was comparatively better than that of the 1/2ABC. Whether these two techniques should be used and the choice between them depend on specific clinical scenarios.

We also validated the performance of 1/2ABC and 2/3SH in small-volume meningiomas. We included this part of analysis based on the premise that quick and simple measurement of meningioma volume is most often needed in two situations in practice. The first is in the process of deciding to adopt SRS. Although predictors of tumor response and complications after SRS are multifactorial, tumor volume alone is an essential component in the decision-making process. In a recent review by Fatima et al., the authors included eight studies published between 1999 and 2018 [25]. They found a strong negative correlation between median tumor volume with clinical improvement and tumor control; increase in size of the tumor was associated with lower probability of clinical improvement and tumor control. Meanwhile, increase in median tumor volume was positively associated with adverse events. This conclusion was generally in line with several previous reports [7–9, 26]. The second is during follow-ups. Patients with small meningiomas may be recommended to undergo observation. The measurement of tumor volume during each follow-up visit, which often occurs in outpatient settings, can benefit by this simple method. Therefore, we performed this subanalysis. Results showed that the general performances of both formula methods in subset 1 and subset 2 were slightly worse. Comparatively, 2/3SH performed better, with such statistical parameters as median, IQR, and minimum/maximum being similar between the entire dataset and subset 1/subset 2. This result confirmed the reliability of the proposed methods, especially 2/3SH, regardless of actual tumor volume.

Theoretically, all calculated volumes should be compared with in vivo measurements, which is the “gold standard” for this study. However, it is impossible in real-world settings. We alternatively used planimetry as the gold standard, as it remains the most accurate among all practical methods. There currently are several software types to carry out planimetry, such as ITK-SNAP and 3D Slicer [21, 27]. As the rationale behind these software types is similar, the choice of the software would not influence the results much. Meanwhile, it should be noted that, during the segmentation process, errors can be introduced. To avoid possible inaccuracies resulting from automatic segmentation, we used manual segmentation instead. Despite being tedious and time-consuming, this process was able to provide the most reliable results to act as the reference standard for our study. Besides, after the initial segmentation, another author (PFY), who has substantial experience in neuro-oncology as well as the use of ITK-SNAP, checked all the segmentations and made modifications when necessary. All these measures were taken to reduce possible errors. Errors are also correlated with slice thickness. Hashiba et al. assessed MR images of 10 patients with brain tumors, and images of each patient had two different slice thicknesses (0.7 and 6.0 mm) [28]. They showed that volumes calculated using MR images with 6.0 mm slice thickness were with acceptable accuracy. Ishi et al. analyzed the correlation between the accuracy of the planimetry with thick-slice MRI and its number of fractions in MRI slices [23]. They were able to conclude that if the number of meningioma-bearing slices was >4, tumor volume could be estimated by planimetry with thick-slice MRI within 10% errors as compared with thin-slice MRI. Considering these findings, we excluded those patients that had the number of tumor-bearing slices ≤4 in the patient selection process. Thus, although slice thickness in our dataset ranged between 4 and 5 mm, the reliability of planimetric volume should not have been compromised.

The basic assumption behind formula methods is that lesions roughly conform to a specific shape, usually an ellipsoid [10]. According to this, we hypothesized that the more the meningioma shape deviated from an ellipsoid, the greater the estimation errors of formula methods are. To test this hypothesis, we introduced the concept of ellipsoidity, which was intuitive and easy to apply for physicians. Unfortunately, our analysis failed to demonstrate a significant correlation between the two variables. This may be explained by the reason that the regularity of meningioma shape had little relevance to the accuracies of formula methods. Another possible reason is that the ellipsoidity defined in our study was not a good index to measure the regularity of tumor shape. Future studies to further address this issue would be interesting and clinically relevant. In addition, as maximum tumor diameter was used to indicate tumor volume in some studies, we evaluated the accuracy of this parameter. Results showed that this parameter could not provide an accurate estimation of tumor volume, which was in accordance with a previous report [23]. As we only reviewed one image series for each patient in this study, the performance of maximum tumor diameter in follow-up images can be further evaluated. Nevertheless, based on current findings, this parameter should be used with great caution, if at all.

There are several limitations to this study. First, this is a retrospective analysis from a single center. As our hospital is a tertiary care teaching institution to which complex cases are referred, meningioma patients with low surgical risks may not be referred to our hospital. This might have resulted in a selection bias. Second, follow-up images were not uniformly available, which prevented us from analyzing the performance of formula methods/maximum tumor diameter on follow-ups. Third, MR images were obtained with different scanners and imaging protocols. Fourth, slice thicknesses of MRI were relatively thick; but as we mentioned above, we reduced the influence of this factor by excluding patients that had the number of tumor-bearing slices ≤4.

## 5. Conclusion

From our study, we conclude that the formula methods 2/3SH and 1/2ABC can estimate meningioma volumes with decent accuracy. Compared with the 1/2ABC method, the 2/3SH method showed slightly better performance, especially in small-volume tumors. Whether these two techniques should be used and the choice between them depend on specific clinical scenarios. Ellipsoidity defined here was not found to be a useful parameter in predicting estimation errors of the formula methods. Furthermore, maximum tumor diameter was not found to be a reliable surrogate for actual meningioma volume and therefore should be used with great caution.

## Figures and Tables

**Table 1:** Parameters of the formula methods.

		1/3ABC	1/2ABC	2/3ABC	1/3SH	1/2SH	2/3SH
Calculated volume (mL)	P_25_	7.565	11.347	15.129	5.713	8.570	11.427
	Median	18.477	27.715	36.953	13.347	20.021	26.695
	P_75_	35.290	52.936	70.581	25.751	38.626	51.502
Volume difference (mL)	P_25_	−15.090	−0.941	3.691	−25.277	−12.187	−1.613
	Median	−7.118	0.640	9.506	−13.045	−6.114	0.098
	P_75_	−3.263	3.817	19.397	−5.736	−2.671	1.880
Percentage volume difference (%)	P_25_	−37.201	−5.801	25.598	−53.540	−30.309	−7.079
	Median	−30.757	3.865	38.486	−49.590	−24.385	0.820
	P_75_	−23.078	15.382	53.843	−45.776	−18.664	8.448
Absolute volume difference (mL)	P_25_	3.377	0.793	3.742	5.736	2.693	0.601
	Median	7.146	2.173	9.645	13.045	6.114	1.734
	P_75_	15.160	5.611	19.482	25.277	12.187	4.136
Absolute percentage volume difference (%)	P_25_	23.167	4.752	25.916	45.776	18.693	3.624
	Median	30.757	9.284	38.725	49.590	24.385	7.790
	P_75_	37.201	17.494	53.993	53.540	30.309	12.833
Spearman rank correlation	*r*_s_	0.987	0.987	0.987	0.994	0.994	0.994
	95% CI of *r*_s_	0.985–0.989	0.985–0.989	0.985–0.989	0.993–0.995	0.993–0.995	0.993–0.995
Intraclass correlation	ICC	0.935	0.976	0.913	0.812	0.962	0.990
	95% CI of ICC	0.925–0.944	0.972–0.980	0.900–0.925	0.785–0.836	0.955–0.967	0.989–0.992
Parameters of Bland-Altman plot	Mean (mL)	−10.716	2.249	15.213	−18.007	−8.689	0.629
	Lower LoA (mL)	−31.975	−13.153	−19.941	−50.885	−25.428	−8.981
	Upper LoA (mL)	10.544	17.651	50.367	14.871	8.049	10.239
	Number out of LoA	34	33	42	41	37	34
	Percent out of LoA (%)	5.015	4.867	6.195	6.047	5.457	5.015

*r*_s_, Spearman rank-correlation coefficient; CI, confidence interval; ICC, intraclass correlation coefficient; LoA, limit of agreement.

**Table 2:** Performance of 1/2ABC and 2/3SH in different subsets.

		Entire set		Subset 1		Subset 2	
		1/2ABC	2/3SH	1/2ABC	2/3SH	1/2ABC	2/3SH
Percentage volume difference (%)	P_25_	−5.801	−7.079	−5.912	−5.849	−6.235	−8.046
	Median	3.865	0.820	4.150	2.807	1.993	0.858
	P_75_	15.382	8.448	19.032	10.886	15.733	9.630
Absolute percentage volume difference (%)	P_25_	4.752	3.624	5.146	4.395	4.133	4.119
	Median	9.284	7.790	12.863	8.477	10.218	8.629
	P_75_	17.494	12.833	20.819	14.882	19.383	13.782
Spearman rank correlation	*r*_*s*_	0.987	0.994	0.925	0.969	0.945	0.972
	95% CI of *r*_*s*_	0.985–0.989	0.993–0.995	0.897–0.945	0.957–0.978	0.931–0.956	0.964–0.978
Intraclass correlation	ICC	0.976	0.990	0.891	0.952	0.932	0.966
	95% CI of ICC	0.972–0.980	0.989–0.992	0.852–0.921	0.933–0.965	0.915–0.946	0.957–0.973
Parameters of Bland-Altman plot	Mean (mL)	2.249	0.629	0.388	0.213	0.377	−0.006
	Lower LoA (mL)	−13.153	−8.981	−2.344	−1.532	−3.561	−2.683
	Upper LoA (mL)	17.651	10.239	3.113	1.952	4.324	2.669
	Number out of LoA	33	34	5	3	11	17
	Percent out of LoA (%)	4.867	5.015	3.448	2.069	3.929	6.071
Diagnostic accuracy*∗* (%)	<10	52.802	63.422	43.448	58.621	49.643	58.929
	<20	79.646	93.658	72.414	88.966	76.429	90.357
	<30	93.510	97.788	88.276	94.483	91.429	96.429

*r*_*s*_, Spearman rank-correlation coefficient; CI, confidence interval; ICC, intraclass correlation coefficient; LoA, limit of agreement. ^∗^Criteria were based on the values of absolute percentage volume difference (APVD).

## Data Availability

The data used to support the findings of this study are available from the corresponding author upon reasonable request
